# Synthesis of Quinolin-2-one Alkaloid Derivatives and Their Inhibitory Activities against HIV-1 Reverse Transcriptase

**DOI:** 10.3390/molecules16097649

**Published:** 2011-09-07

**Authors:** Pi Cheng, Qiong Gu, Wei Liu, Jian-Feng Zou, Yang-Yong Ou, Zhong-Yong Luo, Jian-Guo Zeng

**Affiliations:** 1 National Research Center of Engineering Technology For Utilization of Functional Ingredients From Botanicals, College of Horticulture and Landscape Architecture, Hunan Agricultural University, Changsha 410128, Hunan, China; Email: lwhncs618@163.com (W.L.); zoujf2005@163.com (J.-F.Z.); 2 School of Pharmaceutical Sciences, Sun Yat-Sen University, Guangzhou 510006, Guangdong, China; Email: guqiong@sysu.edu.cn (Q.G.); 3 Hunan Engineering Research Center of Botanical Extract, Changsha410128, Hunan, China; Email: oyy713@163.com (Y.-Y.O.); zhongyongluo@hotmail.com (Z.-Y.L.)

**Keywords:** quinolin-2-one, alkaloid, synthesis, HIV-1 RT, activity

## Abstract

Based on an established common pharmacophore of HIV-1 non-nucleoside reverse transcriptase inhibitors (NNTTIs), a series of quinolin-2-one derivatives were synthesized and assayed for their *in vitro* activities against HIV-1 reverse transcriptase (RT) for the first time. Some of the tested compounds were active against HIV-1 RT. Compounds **4a2** and **4d2** showed inhibitory activities with IC_50_ values of 0.21 and 0.15 μM, respectively, with a mode of interaction with RT residues of the allosteric pocket similar to that of efavirenz.

## 1. Introduction

AIDS (Aquired Immune Deficiency Syndrome), caused by human immunodeficiency virus (HIV), a RNA dependent retrovirus, remains one of the major causes of death in the World. HIV-1 reverse transcriptase (RT) is one of the enzymes crucial for the HIV virus replication cycle. Upon entering a host cell, the single stranded viral RNA is converted to double-stranded DNA catalyzed by RT and then the virus DNA inserts into the genome of the host cell. Inhibition of reverse transcriptase (RT), the HIV-encoded polymerase which directs both RNA and DNA synthesis, has been proven to be one of the most effective ways to block viral multiplication [1,2,3,4,5].

**Figure 1:**
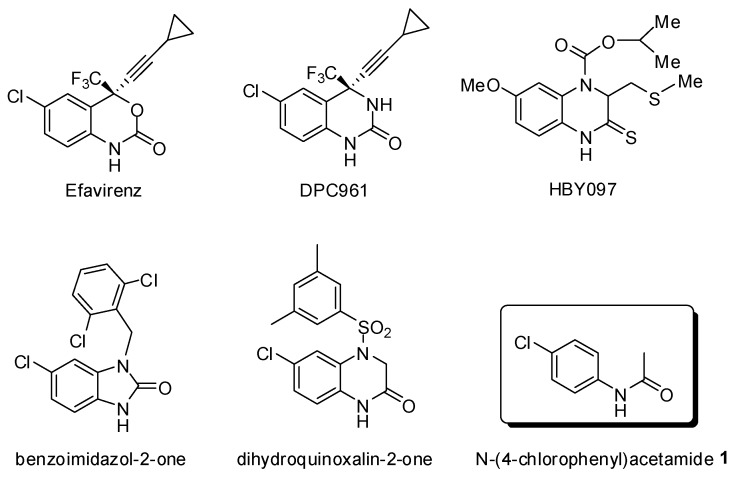
NNRTI pharmacophore *N*-(4-chlorophenyl)acetamide (**1**) and related anti-HIV-1 RT active compounds.

Based on the ready availability of X-ray crystal structures of inhibitor-HIV RT complexes, Freeman and colleagues have identified the common pharmacophore *N*-(4-chlorophenyl)acetamide (**1**) from an array of known non-nucleoside reverse transcriptase inhibitors [6] such as efavirenz, DPC961, HBY097 and the newly reported benzoimidazol-2-one [7] and dihydroquinoxalin-2-one [8] NNTRIs (Figure 1).

**Figure 2:**
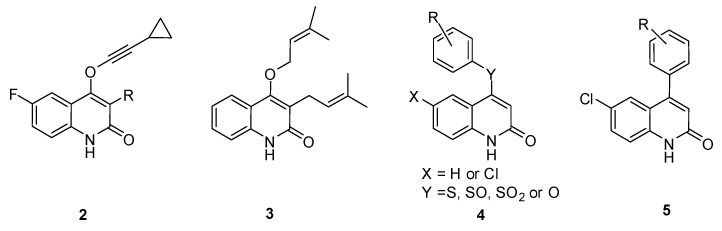
quinolin-2-one NNRTIs and newly designed derivatives.

A series of substituted 2-quinolones (**2**, Figure 2) were synthesized and evaluated as HIV-1 inhibitors in Freeman’s research. As to natural products, a similar quinolin-2-one alkaloid **3** isolated from *Euodia roxburghiana* reported by McCormick also showed inhibitory activity with an IC_50_ of 8 μM in an HIV-1 reverse transcriptase (RT) assay [9].

Based on the pharmacophore established by Freeman, we designed and synthesized a series of 4-substituted quinolin-2-one alkaloids **4** and **5**, as shown in Figure 2. We envisaged that replacement of the cyclopropylethynyloxy moiety with a hydrophobic aromatic ring would retain the inhibitory activity against HIV-1 RT [4,10,11,12,13]. In compounds **4**, the group Y could be oxygen, sulfur, sulfinyl or sulfonyl units, which were flexible linkers to connect the hydrophobic phenyl group with the quinolin-2-one scaffold. To identify the importance of the linker, compounds **5** were synthesized with an aromatic ring directly connected to quinoline-2-one scaffold. In this paper, we report the synthesis of compounds **4** and **5** and the evaluation of the synthesized compounds for their *in vitro* inhibitory activities against HIV-1 RT for the first time.

## 2. Results and Discussion

### 2.1. Chemistry

**Scheme 1:**
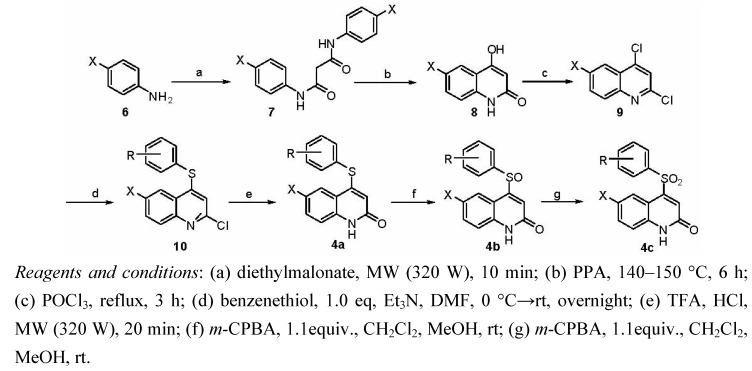
Synthesis of target compounds **4a**–**4c**.

**Scheme 2:**
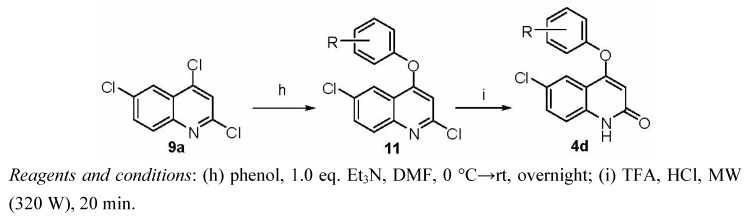
Synthesis of target compounds **4d**.

The syntheses of compounds **4** is summarized in Scheme 1 and Scheme 2, respectively. 3-Hydroxy-quinolin-2-one (**8**, Scheme 1) has been the subject of intensive synthetic studies for a long time. Among the synthetic strategies used is the Friedel-Crafts reaction [6]. Freeman reported that a treatment with aniline or 4-chloroaniline **6** and excess of diethyl malonate in diphenyl ether at 250 °C for 24 h afforded cyclized compound **8** directly. But in our study, target compound **8** was not obtained under these conditions and only some amide intermediates could be detected by HPLC-ESIMS. We thus irradiated a mixture of aniline and diethylmalonate (2:1 molar ratio) under microwaves and prepared corresponding dimalonamide **7** in good yield. Heating *N*,*N*’-di(4-chlorophenyl)malonamide in polyphosphoric acid (PPA) at 140–150 °C afforded the 4-hydroxy-2-quinolone **8** (Scheme 1) [14]. Refluxing compound **8** in POCl_3_ provided 2,4,6-trichloroquinoline **9** in good yield. A phenylthio group was then installed on the quinoline ring to afforded compounds **10**, which were converted to target compounds **4a** through a microwave assisted hydrolysis in mixture of TFA and HCl [15]. Compounds **4a** were oxidized to target compounds **4b** as racemates, and these were then converted to compounds **4c** by reaction with another 1.1 equiv. of 3-chlorobenzoperoxoic acid (*m-*CPBA).

The synthesis of compounds **4d** shared the same synthetic precursor 2,4,6-trichloroquinoline **9** (Scheme 2). Through treatment of compound **9a** with various phenols followed by hydrolysis in acid mixtures, compounds **4d** were synthesized in moderate yields using the conditions described above without need for further optimization.

**Scheme 3:**
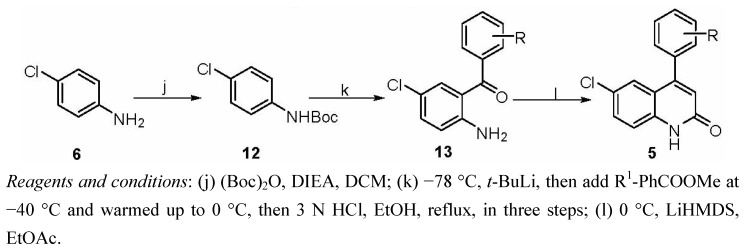
Synthesis of target compounds **5**.

The synthetic route of target compounds **5** was summarized in Scheme 3. The synthesis of **5** by various methods has been reviewed. The majority of compounds **13** were prepared by the reaction of aryl esters with *ortho*-lithiated Boc-protected 4-chloroaniline via the formation of the dianion species with *t*-BuLi (2.2 equiv) followed by deprotection of the Boc group (Scheme 3) [16,17]. A tandem amidation/Knoevengel condensation of compounds **13** with ethyl acetate gave compounds **5** in good yields.

### 2.2. Enzymatic Activities

Compounds **4a**–**4d** were tested in RT inhibition assays and proved to be active as inhibitors of HIV-1 RT (Table 1). The enzymatic data highlighted that derivative **4a2** (IC_50_ = 0.21 μM) and **4d2** (IC_50_ = 0.15 μM) turned out to be more active than **4a3** (IC_50_ = 13 μM), respectively, and suggested that the presence of a chlorine substituent at C-6 may lead to an increase of potency and influence the affinity to the enzyme. This finding is in agreement with the *N*-(4-chlorophenyl)acetamide pharmacophore established by Freeman, and new type of anti-HIV-1 RT compounds could be designed based on the pharmacophore model.

**Table 1:** HIV-1 RT inhibitory activities of compounds 4–5. ^a^

**Compounds**	X	Y	R	IC_50_ (μM) ^b,c^
**4a1**	Cl	S	H	5.6
**4a2**	Cl	S	3,5-CH_3_	0.21
**4a3**	H	S	3,5-CH_3_	18
**4b1**	Cl	SO	H	8.9
**4b2**	Cl	SO	3,5-CH_3_	2.6
**4c1**	Cl	SO_2_	H	49
**4c2**	Cl	SO_2_	3,5-CH_3_	10
**4d1**	Cl	O	H	3.0
**4d2**	Cl	O	3,5-CH_3_	0.15
**5a**	Cl	/	H	>100
**5b**	Cl	/	2-F	>100
**5c**	Cl	/	2-Cl	>100

^a^ All values are the mean of two independent experiments; ^b^ The inhibition of recombinant HIV-1 RT activity was performed with a commercially available ELISA kit (Roche) according to the instructions of the manufacturer. IC_50_ = effective concentration that inhibits 50% of HIV-1 RT; ^c^ Efavirenz: an HIV-1 RT inhibitor used as positive control. IC_50_ = 6 nM.

In addition, the type of linker which connected the aromatic ring with the quinoline scaffold might be crucial for potent anti-HIV-1 RT activity in the title compounds. Comparing compounds **4a2** (IC_50_ = 0.21 μM), **4b2** (IC_50_ = 2.6 μM) and **4c2** (IC_50_ = 10 μM), a decrease of activity against RT could be observed as the degree of oxidation of the sulfur atom increased. The role of the substituents of the phenyl ring on the biological activities was also investigated, as shown in Table 1. In compounds **4a1** (IC_50_ = 5.6 μM) and **4a2** (IC_50_ = 0.21 μM), two hydrophobic methyl group on the phenyl ring led to potent enzyme activity, and may contribute to the ability to bind with the hydrophobic site of RT. In contrast, compounds **5a**–**5c** exhibited obviously decreased activities towards the enzyme. We envisagd that hydrophobic interaction ability was influenced by the rigid conjugation system between the phenyl ring and the quinolin-2-one scaffold.

### 2.3. Docking Studies

**Figure 3:**
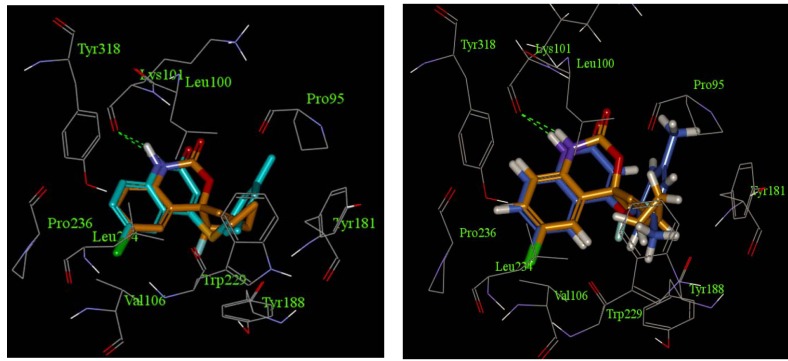
CDOCK-modeled binding mode of **4a2** (left) and **4d2** (right) in comparison with the crystal structure (1FK9 in PDB) of efavirenz (orange colored carbon atoms). The key hydrogen bond is illustrated with green lines.

To investigate the binding mode of the synthesized quinolin-2-ones, computational modeling was performed using CDOCK. The CHARMM force field was used for the energy minimizations in the docking process. The coordinates of the RT-wfavirenz complex (PDB code: 1FK9) [12,18] were downloaded, and the molecular structure of the most potent compounds **4a2** and **4d2** were docked into its active site. The best docked conformation of compounds **4a2** and **4d2** were shown in Figure 3, together with the experimental position of efavirenz and the 3D common-feature pharmacophore model. Compounds **4a2** and **4d2** interact with RT residues of the allosteric pocket in a fashion similar to known NNRTIs. The *p*-chloroaniline moiety has a hydrophobic contact with the surrounding residues such as Tyr318, Pro236, Leu234 and Val106. The carboxamide group of the molecule interacts with the main-chain of Lys101 by hydrogen bond interactions. The 3,5-dimethylphenyl fragment occupies a hydrophobic pocket consisting of Pro95, Tyr181, Tyr188 and Trp229, which is occupied by the cyclopropyl-propynyl of efavirenz in the complex. One of the two methyl groups on the benzene ring adopts a similar orientation to the cyclopropyl group of efavirenz.

## 3. Experimental

### 3.1. Chemistry

#### 3.1.1. Instruments and Materials

Column chromatography silica gel (200-300 mesh) and TLC plate (Qingdao Meijin Chemical Inc.; Qingdao; China); MS data were obtained on an AutoSpec-3000 or Agilent HPLC-ESIMS; ^1^H-NMR spectra were recorded on Bruker 300M or DRX-500 spectrometers and chemical shifts were given in *δ* with TMS as an internal reference. All the reagents are commercial available. Tetrahydrofuran (THF) was refluxed over Na before use.

#### 3.1.2. General Procedure for Preparation of Compounds **4**

##### 3.1.2.1. Synthesis of Intermediates **7**

Aniline or 4-chloroaniline (100 mmol), and diethyl malonate (50 mmol) were properly mixed in a 250 mL beaker. The obtained mixture was irradiated in a microwave oven at the power of 320 W for 5 min. After irradiation, the crude product was recrystallised in ethanol to afford the desired product.

*bis(4-Chlorophenyl)malonamide* (**7a**): yield 95%, white solid, ESIMS: *m/z*: 323 (M+H)^+^

*Diphenylmalonamide* (**7b**): yield 91%, white solid, ESIMS: *m/z*: 255 (M+H)^+^

##### 3.1.2.2. Synthesis of Intermediates **8**

A mixture of **7a** or **7b** (2.0 mmol) with 5–6 times by weight polyphosphoric acid were stirred in an oil bath at 140–150 °C for 6 h. Then the mixture was cooled, diluted with water and the resultant gum solidified by standing over night. The solid was filtered and dried in air. It was recrystallised from ethanol to afford corresponding hydroxyquinolones as creamy crystals.

*6-Chloro-4-hydroxyquinolin-2(1H)-one* (**8a**): white solid, yield: 82%, ^1^H-NMR (300 MHz, DMSO-*d*_6_, *δ* ppm): 5.80 (s, 1H), 7.68 (s, 1H), 7.27 (dd, 1H, *J* *=* 8.6, 4.8 Hz), 7.53 (d, 1H, *J* *=* 8.6 Hz), 11.35 (brs, 1H, NH).

*4-Hydroxyquinolin-2(1H)-one* (**8b**): white solid, yield: 85%, ^1^H-NMR (300 MHz, DMSO-*d*_6_, *δ* ppm): 5.83 (s, 1H), 7.51–7.68 (m, 3H), 7.23 (d, 1H, *J* *=* 8.0 Hz), 11.33 (brs, 1H, NH).

##### 3.1.2.3. Synthesis of Intermediates **9**

A mixture of **8** (84.47 mmol) in POCl_3_ (150 mL) was heated under reflux for 3h. After cooling down, the mixture was slowly added to crushed ice while shaking. The precipitate was collected by filtration and washed several times with water. The product was dried under vacuum overnight to give the products as white solid which are pure enough for further use.

*2,4,6-Trichloroquinoline* (**9a**): white solid, yield 70%, ^1^H-NMR (500 MHz, CDCl_3_, *δ* ppm): 8.00 (s, 1H), 7.94 (d, *J =* 9.0 Hz, 1H), 7.66 (d, *J =* 9.0 Hz, 1H), 7.56 (s, 1H); ESIMS: *m/z*: 232 (M+H)^+^.

*2,4-Dichloroquinoline* (**9b**): white solid, yield 66%, ^1^H-NMR (500 MHz, CDCl_3_, *δ* ppm): 8.16 (d, *J =* 8.0 Hz, 1H), 8.01 (d, *J =* 8.0 Hz, 1H), 7.80 (t, *J =* 8.0 Hz, 1H), 7.65 (t, *J =* 8.0 Hz, 1H), 7.47 (s, 1H); ESIMS: *m/z*: 198 (M+H)^+^.

##### 3.1.2.4. Synthesis of Intermediates **10**

To a solution of **9a** or **9b** (10.0 mmol) in DMF (10 mL) was added Et_3_N (2.78 mL, 20 mmol) and a solution of thiol (10 mmol) in DMF (10 mL) dropwise at 0 °C. The reaction was warmed up after 10 min and stirred overnight at rt. 200 mL of EtOAc was added to the mixture and washed with water and brine successively. The organic layer was separated and dried over Na_2_SO_4_. The product was purified on a column after removal of the solvent under vacuum. In most of cases, less than 7% of dithio-substituted quinoline was formed.

*2,6-Dichloro-4-(phenylthio)quinoline* (**10a**): amorphous powder, yield: 69%. ^1^H-NMR (300 MHz, CDCl_3_, *δ* ppm): 8.11 (s, 1H), 7.88–7.50 (m, 2H), 7.18–7.09 (m, 5H), 6.72 (s, 1H); ESIMS: *m/z*: 306 (M+H)^+^.

*2,6-Dichloro-4-(3,5-dimethylphenylthio)quinoline* (**10b**): amorphous powder, yield: 69%. ^1^H-NMR (300 MHz, CDCl_3_, *δ* ppm): 8.13 (s, 1H), 7.88–7.50 (m, 2H), 7. 24 (s, 2H), 7.19 (s, 1H), 6.62 (s, 1H), 2.34 (s, 6H); ESIMS: *m/z*: 334 (M+H)^+^.

*2-chloro-4-(3,5-dimethylphenylthio)quinoline* (**10c**): amorphous powder, yield: 59%. ^1^H-NMR (300 MHz, CDCl_3_, *δ* ppm): 8.23 (d, *J =* 8.0 Hz, 1H), 7.99–7.57 (m, 3H), 7.23 (s, 1H), 7.21 (s, 1H), 7.16 (s, 1H), 6.63 (s, IH), 2.33 (s, 6H); ESIMS: *m/z*: 282 (M+H)^+^.

##### 3.1.2.5. Synthesis of Target Compounds **4a**

A solution of compound **10** (0.50 mmol) in TFA (2 mL) and HCl (6 N, 2 mL) was heated under microwave radiation at 120 °C for 20 min. After cooling down, water (20 mL) was added. The precipitate was collected by filtration and washed with water several times. The product was air dried (71–85%) and was pure enough without further purification. The solubility of the products in organic solvents is poor.

*6-Chloro-4-(phenylthio)quinolin-2(1H)-one* (**4a1**): amorphous powder, yield: 71%. ^1^H-NMR (300 MHz, DMSO-*d_6_*, *δ* ppm): 11.13 (bs, 1H), 7.81 (s, 1H), 7.68–7.30 (m, 2H), 7.08–6.89 (m, 5H), 5.72 (s, 1H); ESIMS: *m/z*: 288 (M+H)^+^; HRESIMS: calc for C_15_H_11_ClNOS [M+H]^+^ 288.0250, found 288.0261.

*6-Chloro-4-(3,5-dimethylphenylthio)quinolin-2(1H)-one* (**4a2**): amorphous powder, yield: 77%. ^1^H-NMR (300 MHz, DMSO-*d_6_*, *δ* ppm): 11.38 (bs, 1H)7.76 (s, 1H), 7.68–7.30 (m, 2H), 7. 22 (s, 2H), 7.18 (s, 1H), 5.82 (s, 1H), 2.34 (s, 6H); ESIMS: *m/z*: 316 (M+H)^+^; HRESIMS: calc for C_17_H_15_ClNOS 316.0563, found 316.0547.

*4-(3,5-dimethylphenylthio)quinolin-2(1H)-one* (**4a3**): amorphous powder, yield: 85%. ^1^H-NMR (300 MHz, DMSO-*d_6_*, *δ* ppm): 11.66 (bs, 1H), 7.88 (d, *J =* 8.0 Hz, IH), 7.89–7.43 (m, 3H), 7.251 (s, 2H), 7.13 (s, 1H), 5.88 (s, IH), 2.32 (s, 6H); ESIMS: *m/z*: 282 (M+H)^+^; HRESIMS: calc for C_17_H_16_NOS [M+H]^+^ 282.0953, found 282.0950.

##### 3.1.2.6. Synthesis of Target Compounds **4b**

To a solution of compound **4a** (0.25 mmol) in a mixture of CH_2_Cl_2_ and MeOH (50 mL) was added 1.1 equiv. of 3-chlorobenzoperoxoic acid. After stirring overnight, water (50 mL) was added. The mixture was extracted with CH_2_Cl_2_ and evaporated to give a residue which were further purified on silica gel column to give compounds **4b** (66–75%).

*6-Chloro-4-(phenylsulfinyl)quinolin-2(1H)-one* (**4b1**): amorphous powder, yield: 66%. ^1^H-NMR (300 MHz, DMSO-*d_6_*, *δ* ppm): 11.85 (bs, 1H), 7.91 (s, 1H), 7.88–7.47 (m, 7H), 5.92 (s, 1H), 2.39 (s, 6H); ESIMS: *m/z*: 304 (M+H)^+^; HRESIMS: calc for C_15_H_11_ClNO_2_S [M+H]^+^ 304.0199, found 304.0208.

*6-Chloro-4-(3,5-dimethylphenylsulfinyl)quinolin-2(1H)-one* (**4b2**): amorphous powder, yield: 75%. ^1^H-NMR (300 MHz, DMSO-*d_6_*, *δ* ppm): 11.40 (bs, 1H), 7.96 (s, 1H), 7.88–7.55 (m, 2H), 7. 47 (s, 1H), 7. 42 (s, 1H), 7.40 (s, 1H), 6.01 (s, 1H), 2.32 (s, 6H); ESIMS: *m/z*: 332 (M+H)^+^; HRESIMS: calc for C_17_H_15_ClNO_2_S [M+H]^+^ 332.0512, found 332.0509.

##### 3.1.2.7. Synthesis of Target Compounds **4c**

To a solution of compound **4b** (0.2 mmol) in a mixture of CH_2_Cl_2_ and MeOH (50 mL) was added 1.1 equiv. 3-chlorobenzoperoxoic acid. After stirring overnight, 50 mL of water was added. The mixture was extracted with CH_2_Cl_2_ and evaporated to give a residue which were further purified on silica gel column to give compound **4b** (60–66%).

*6-Chloro-4-(phenylsulfonyl)quinolin-2(1H)-one* (**4c1**): amorphous powder, yield: 64%. ^1^H-NMR (300 MHz, CDCl_3_): 11.51 (bs, IH), 8.10 (s, 1H), 7.91–7.49 (m, 5H), 7.39 (d, *J* *=* 8.0 Hz, 1H), 7.29 (d, *J* *=* 8.0 Hz, 1H), 6.12 (s, 1H); ESIMS: *m/z*: 320 (M+H)^+^; HRESIMS: calc for C_15_H_11_ClNO_3_S [M+H]^+^ 330.0148, found 320.0154.

*6-Chloro-4-(3,5-dimethylphenylsulfonyl)quinolin-2(1H)-one* (**4c2**): amorphous powder, yield: 60%. ^1^H-NMR (300 MHz, CDCl_3_, *δ* ppm): 11.86 (bs, IH), 8.00 (s, 1H), 7.98–7.69 (m, 2H), 7.57 (s, 2H), 7.39 (s, 1H), 5.95 (s, 1H), 2.35 (s, 6H); ESIMS: *m/z*: 348 (M+H)^+^; HRESIMS: calc for C_17_H_15_ClNO_3_S [M+H]^+^ 348.0461, found 348.0470.

##### 3.1.2.8. Synthesis of Target Compounds **4d**

To a solution of **9a** (10.0 mmol) in DMF (10 mL) was added Et_3_N (2.78 mL, 20 mmol) and a solution of phenol (10 mmol) in DMF (10 mL) dropwise at 0 °C. The reaction was warmed up after 10 min and stirred for 24h at rt. 200 mL of EtOAc was added to the mixture and washed with water and brine successively. The organic layer was separated and dried over Na_2_SO_4_. The product **11** was purified on column after removal of solvent under vacuum with yield of 75–86%. A solution of compound **11** (0.50 mmol) in TFA (2 mL) and HCl (6 N, 2 mL) was then heated under microwave radiation at 120 °C for 20 min. After cooling down, 20 mL of water was added. The precipitate was collected by filtration and washed with water for several times. The product was air dried (71–85%) and was pure enough without further purification.

*6-Chloro-4-phenoxyquinolin-2(1H)-one* (**4d1**): amorphous powder, yield: 71%. ^1^H-NMR (300 MHz, DMSO-*d_6_*, *δ* ppm): 11.94 (bs, 1H), 7.71 (s, 1H), 7.60–7.10 (m, 2H), 7.01–6.67 (m, 5H), 5.70 (s, 1H); ESIMS: *m/z*: 272 (M+H)^+^; HRESIMS: calc for C_15_H_11_ClNO_2_ [M+H]^+^ 272.0478, found 272.0470.

*6-Chloro-4-(3,5-dimethylphenoxy)quinolin-2(1H)-one* (**4d2**): amorphous powder, yield: 77%. ^1^H-NMR (300 MHz, DMSO-*d_6_*, *δ* ppm ): 11.64 (bs, 1H)7.70 (s, 1H), 7.65–7.31 (m, 2H), 6.89 (s, 2H), 6.93 (s, 1H), 5.72 (s, 1H), 2.33 (s, 6H); ESIMS: *m/z*: 300 (M+H)^+^; HRESIMS: calc for C_17_H_15_ClNO_2_ [M+H]^+^ 300.0791, found 300.0775.

#### 3.1.3. General Procedure for Preparation of Compounds **5**

##### 3.1.3.1. Synthesis of Intermediate **12**

4-Chloroaniline (19.2 g, 0.1 mol), DIEA (0.12 mol) and toluene (40 mL) were placed in a 100 mL flask and stirred at rt, then Boc_2_O (26 g, 0.12 mol) was added, and the solution was stirred at rt until the completion of the reaction monitored by TCL. Then the solution was poured into water and extracted with DCM and then the organic layer was washed by water and dried over anhydrous Na_2_SO_4_. Then the organic solvent was removed under vacuum to give crude product which was further purified on Si gel column chromatography and eluted with petroleum ether (PET)-EtOAc (6:1) to give the compoun*d* *tert-butyl 4-chlorophenylcarbamate* (**12**) 21.3 g, white solid, yield 94%. ^1^H-NMR (CDCl_3_, *δ* ppm): 7.31–7.22 (m, 4H), 6.54 (brs, 1H, NH), 1.51 (s, 9H, CH_3_); positive FABMS *m/z* (%): 227 [M]^+^ (60), 172 (83), 57 (100).

##### 3.1.3.2. Synthesis of Compound **13**

Compound **12** was taken as an example. To a 100 mL flask, Boc_2_O (11.4 g, 50 mmol) and anhydrous THF (30 mL) were added under an atmosphere of N_2_. The solution was stirred and cooled to −78 °C, then t-BuLi solution in THF (110 mmol) was added dropwise under N_2_. The solution was kept at −78 °C under stirring for 1 h and then warmed up to −40°C. Methyl benzoate (60 mmol, in 10 mL THF) was added dropwise under N_2_ in 15 min. The solution was kept in −40 °C for another 1 h and warmed up to rt. The solution was poured into ice cooled NH_4_Cl solution and extracted with EtOAc. The combined organic layer was washed with brine and dried over anhydrous Na_2_SO_4_ and then evaporated to give crude *N*-Boc protected **13**. Without further purification, the crude product was dissolved in 30 mL EtOH, and then 3 mol/L HCl solution (10 mL) was added. The mixture was refluxed until the completion of the reaction. Then the solution was adjusted to pH 8 using saturated NaHCO_3_ solution and extracted with DCM successively. The organic layer was washed with brine and dried over anhydrous Na_2_SO_4_ and then evaporated to give a residue which was further purified on Si CC to give compound **13** (3.46 g, yield: 30%).

*(2-Amino-5-chlorophenyl)(phenyl)methanone* (**13a**): ^1^H-NMR (DMSO-*d*_6_, *δ* ppm): 7.64–7.33 (m, 7H), 6.99 (d, *J =* 8.8 Hz, 1H), 6.08 (brs, 2H, NH_2_); positive FABMS *m/z* (%): 232 [M+1]^+^ (100).

*(2-Amino-5-chlorophenyl)(2-fluorophenyl)methanone* (**13b**): yellow amorphous powder, yield 27%, ^1^H-NMR (DMSO-*d*_6_, *δ* ppm): 7.50–6.15 (m, 6H), 6.67 (d, *J =* 8.8 Hz, 1H), 6.38 (brs, 2H, NH_2_); positive FABMS *m/z* (%): 250 [M+1]^+^ (100). 249 [M]^+^ (100).

*(2-Amino-5-chlorophenyl)(2-chlorophenyl)methanone* (**13c**): yellow amorphous powder, yield 31%, ^1^H-NMR (DMSO-*d*_6_, *δ* ppm): 7.46–7.11 (m, 6H), 6.68–6.65 (m, 1H), 6.47 (brs, 2H, NH_2_); positive FABMS *m/z* (%): 266 [M+1]^+^ (100).

##### 3.1.3.3. Synthesis of Compounds **5**

Compound **5a** was taken as an example. A 30 mL, 2-necked flask was charged with compound **13a** (1 mmol, 231 mg) and THF (5 mL). The resulting solution was cooled to 0 °C and LiHMDS (6 mL, 1M in THF) was added over 5 min. The internal temperature was controlled <5 °C for 10 min, then EtOAc (144 mg, 2 mmol) was added over 1 min. The reaction solution was allowed to warm up to rt and stirred at rt for 2h. Then water (5 mL) was added, and the reaction mixture was stirred at rt for another 24 h. The solution was poured into 50 mL ice cooled water, and a water suspension formed. The suspension was filtered and the solid was further purified on Si CC eluted with CHCl_3_/CH_3_OH (97:3) to give white amorphous powder **5a** (212 mg, yield: 83%).

*6-Chloro-4-phenylquinolin-2(1H)-one* (**5a**): white amorphous powder, yield:70% ^1^H-NMR (DMSO-*d*_6_, *δ* ppm): 12.02 (s, 1H, NH), 7.60–7.39 (m, 7H), 7.26 (s, 1H), 6.45 (s, 1H); positive FABMS *m/z* (%): 256 [M+1]^+^ (50), 73 (100). HRESIMS: calc for C_15_H_11_ClNO [M+H]^+^ 256.0529, found 256.0548.

*6-Chloro-4-(2-fluorophenyl)quinolin-2(1H)-one* (**5b**): white amorphous powder, yield: 77%, ^1^H-NMR (CDCl_3_-CD_3_OD, *δ* ppm): 8.16–8.10 (m, 3H), 8.01–7.96 (m, 3H), 7.89–7.87 (m, 2H); EI-MS *m/z* (%): 275 [M+2]^+^ (6), 274 [M+1]^+^ (4), 273 [M]^+^ (18), 246 (17), 210 (65), 131 (100), 117 (50), 105 (21), 91 (84): HRESIMS: calc for C_15_H_10_ClFNO [M+H]^+^ 274.0435, found 274.0448.

*6-Chloro-4-(2-chlorophenyl)quinolin-2(1H)-one* (**5c**): white amorphous powder, yield: 80%, ^1^H-NMR (DMSO-*d*_6_, *δ* ppm): 12.11 (s, 1H, NH), 7.67–7.40 (m, 4H), 6.84 (d, *J =* 2.2 Hz, 1H), 6.47 (s, 1H); EI-MS *m/z* (%): 293 [M+4]^+^ (4), 293 [M+3]^+^ (3), 291 [M+2]^+^ (10), 289 [M]^+^ (20), 167 (30), 149 (100); HRESIMS: calc for C_15_H_10_C_l2_NO [M+H]^+^ 290.0139, found 290.0151.

### 3.2. General Procedure for HIV-1 RT Inhibitory Assay

HIV-1 reverse transcriptase (RT) activity was measured using a commercially available ELISA RT kit (Roche) according to the instructions of the manufacturer. The compounds were incubated with DIG-labeled reaction mixture at 37 °C for 2 h, then anti-DIG-POD solution was added, followed by substrate ABTS. Efaviren was used as a positive control. The absorbency at 405 nm/490 nm (A405/490) was read on Bio-Tek ELx 800 ELISA reader.

### 3.3. General Procedures for Docking Study

CDOCK was used for the docking study. The CHARMM force field was used for the energy minimizations in the docking process. The X-ray crystal structure of reverse transcriptase complexed with efavirenz was retrieved from PDB (PDB code: 1FK9). The active site was defined as all residues within 6.5 Å radius of the cocrystallized efavirenz. Starting from the ligand configuration, a set of 10 different orientations was randomly generated and ranked according to their binding free energy. The docking mode was chosen on the basis of binding affinity rank.

## 4. Conclusions

In summary, a group of quinoneline-2-one analogues with activity against HIV-1 RT were synthesized based on the SAR established by Freeman and known crystal structures. The SAR and docking studies of the titled compounds were analyzed. Analogues **4a2** and **4d2** demonstrated the most activity among these analogues with a similar mode of interaction with RT residues of the allosteric pocket as known NNRTIs. Further structure modifications and cell-based *in vitro* anti-HIV-1 assay studies of these quinolin-2-one derivatives will be reported in due course.
